# What Property of the Contour of a Deforming Region Biases Percepts toward Liquid?

**DOI:** 10.3389/fpsyg.2017.01014

**Published:** 2017-06-15

**Authors:** Takahiro Kawabe

**Affiliations:** NTT Communication Science Laboratories, Nippon Telegraph and Telephone CorporationAtsugi, Japan

**Keywords:** perceptual transparency, image deformation, material perception, shape perception, non-rigidity

## Abstract

Human observers can perceive the existence of a transparent surface from dynamic image deformation. They can also easily discriminate a transparent solid material such as plastic and glass from a transparent fluid one such as water and shampoo just by viewing them. However, the image information required for material discrimination of this sort is still unclear. A liquid changes its contour shape non-rigidly. We therefore examined whether additional properties of the contour of a deformation-defined region, which indicated contour non-rigidity, biased percepts of the region toward liquid materials. Our stimuli had a translating circular region wherein a natural texture image was deformed at the spatiotemporal deformation frequency that was optimal for the perception of a transparent layer. In Experiment 1, we dynamically deformed the contour of the circular region and found that large deformation of the contour biased the percept toward liquid. In Experiment 2, we manipulated the blurriness of the contour and observed that a strongly blurred contour biased percepts toward liquid. Taken together, the results suggest that a deforming region lacking a discrete contour biases percepts toward liquid.

## Introduction

Recognizing materials is a complex but important task for the visual system. For example, by differentiating solid from liquid materials, humans can adaptively control their actions toward an object, such as how to grasp it (Paulun et al., 2016), or whether they can walk on it or eat it. Recent studies have clarified that both shape (Paulun et al., 2015; van Assen and Fleming, 2016) and motion (Kawabe et al., 2015b) cues are effective for discriminating liquid viscosity.

In addition to liquid viscosity, dynamic image deformation contributes to the perception of a transparent liquid layer. Specifically, a specific band in the spatiotemporal frequency of image deformation is a critical cue to perceiving the existence of a transparent liquid-like layer (Kawabe et al., 2015a). Moreover, the magnitude of image deformation is possibly a decisive factor in discriminating a hot air flow from a water flow (Kawabe and Kogovšek, 2017).

Although considerable efforts have been made to advance our understanding of how the visual system discriminates transparent liquid materials (Kawabe et al., 2015a; Kawabe and Kogovšek, 2017), it is still not clear how an observer discerns transparent “liquid” materials such as water and shampoo from “solid” materials such as plastic and glass.

Physically, the contour shape of a transparent liquid material flexibly changes across time, while that of a transparent solid material tends to be preserved across time, though both of the materials often produce non-rigid image deformation due to light refraction. That is, as we experience in our daily life, a water drop sliding along a glass window surface non-rigidly changes its contour shape along its trajectory, while a transparent glass marble sliding on a tabletop preserves its shape.

In the present study, we examine how the non-rigid properties of the contour of a deforming region bias the percept of the region toward liquid.

Previous studies have found some properties of an object's contour that affect the interpretation of a surface. Nefs (2008) reported that the perception of shape from shading was altered when both the specular highlights and occluding contours were eliminated from the stimuli. Knill (1992) showed that the deformation of a contour shape of a uniform surface can affect the transparency judgment of the surface. Moreover, Todorović (2014) demonstrated the role of contour shape in the perception of shape from shading. A different line of research (Marlow and Anderson, 2015; Marlow et al., 2015) showed that such manipulation of contour shape influenced the assessment of surface properties as either glossy or matte. In this way, the manipulation of contour shape properties affects the visual interpretation of surface properties such as shape, lightness, and reflection.

Some studies have already proposed some contour properties that determine the rigidity of an object and/or a surface. For example, it was reported that the shape representation of a dynamic circular array of dots affected the non-rigidity perception of the array (Cohen et al., 2010). The shape from motion cue also plays an important role in determining the perception of the rigidity of an object (Jain and Zaidi, 2011). Moreover, the phase difference present in the oscillation of the “Packman” inducers can elicit the elasticity impression of a surface defined by subjective contours (Masuda et al., 2013).

We examined whether contour properties that are likely to enhance the interpretation of non-rigidity of a contour biased the percept of a deforming region toward liquid. In Experiment 1, we examined the effect of the non-rigid deformation of the contour of a deforming region on the percept of a material toward liquid. In Experiment 2, we explored the effect of spatially blurring the contour of a deforming region on the percept of a deforming region toward liquid. It has been shown that spatially blurring the contour of a gray patch against a textured background produced the impression of fog or smoke (Anderson et al., 2006). We assume here that spatially blurring the contour of a deforming region degraded the perception of surface geometry (Nandakumar et al., 2011), and the lack of the perceived surface geometry along a contour would facilitate the interpretation of a non-rigid material. It was thus expected that the image blurring of a contour would bias the percept of a material toward liquid. Based on the results of the two experiments, we discuss how the human visual system uses contour information as a cue to determine the material type of a deforming region.

## Experiment 1

### Purpose

The purpose of this experiment was to check whether applying a non-rigid deformation to the contour of a deforming region biased the percept of the region toward liquid. It was predicted that observers would report the stronger impression of liquid as the magnitude of non-rigid contour deformation increased.

### Method

#### Observers

A group of 10 observers consisting of six females and four males participated in this study. Their mean age was 30 years with standard deviation of 5.1 years. They were unaware of the specific purpose of the experiments. They reported having normal or corrected-to-normal visual acuity, and normal color vision. Participants were recruited from outside of the laboratory and paid for their participation. Ethical approval for this study was obtained from the ethical committee at Nippon Telegraph and Telephone Corporation (H28-008 by NTT Communication Science Laboratories Ethical Committee). The experiments were conducted according to the principles laid down in the Helsinki Declaration. Written informed consent was obtained from all participants.

#### Apparatus

Stimuli were presented on a 21-inch CRT monitor (GDM-F500R, Sony) with a resolution of 1024 × 768 pixels and a refresh rate of 60 Hz. A computer (Mac Pro, Apple Inc.) controlled stimulus presentation and data collection with Psychopy v1.83 (Peirce, 2007, 2009).

#### Stimuli

**Figure 1:**
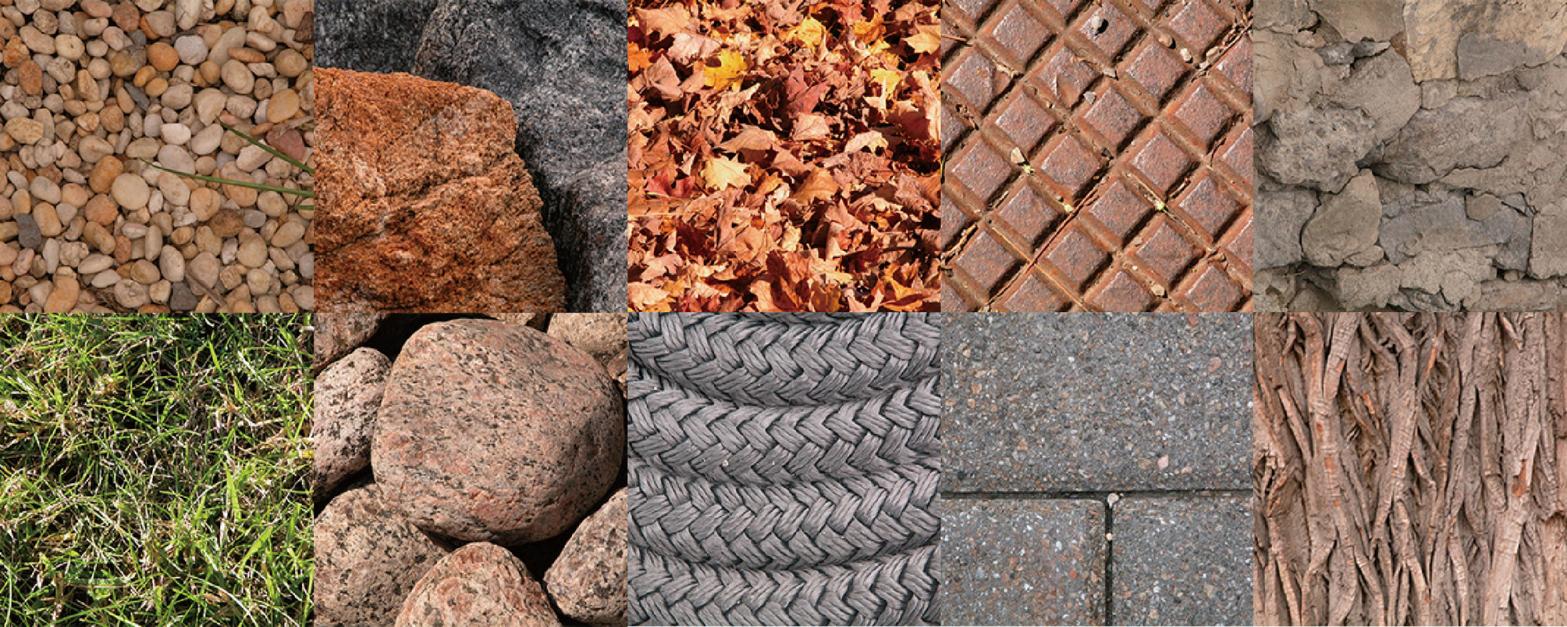
Ten images used as background images in this study.

**Figure 2:**
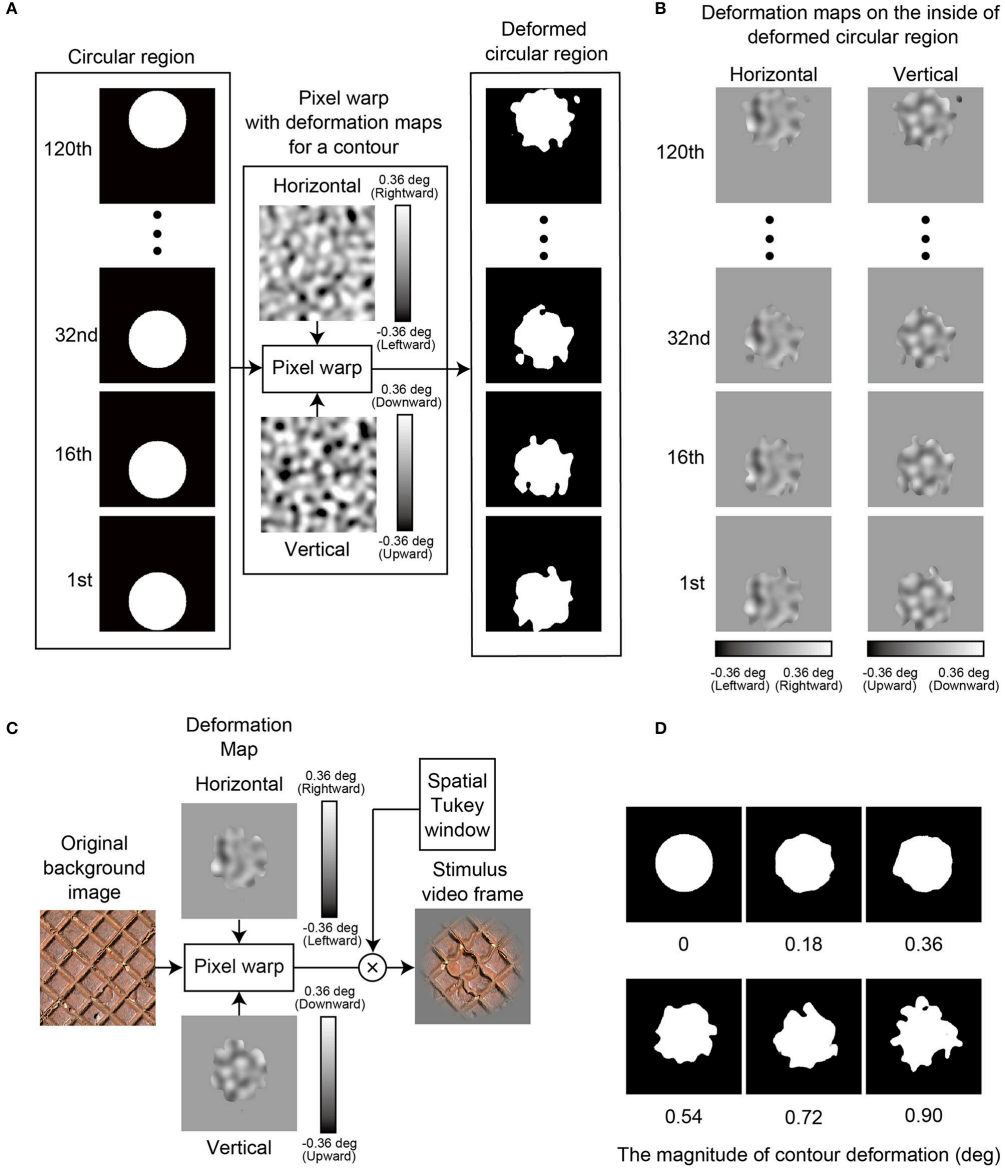
How to make a stimulus image sequence: Experiment 1. **(A)** The method to deform the contour shape of a circular region at the contour deformation stage. As shown by the vertical luminance bars, a grayscale value in a deformation map denotes the magnitude of a motion vector by which a pixel in a contour of the circular region was moved. Because a circular region was translated upward (the deformation map was kept static), the contour of the circular region was deformed by motion vectors of different magnitude across frames. “Pixel warp” refers to a pixel warp method (Glasbey and Mardia, 1998). **(B)** Deformation maps as used in the image deformation stage. **(C)** The method to deform a background image within the deformed circular region at the image deformation stage. As shown by the vertical luminance bars, a grayscale value in a deformation map denotes the magnitude of a motion vector by which a pixel in a background image was moved. Because the deformation map was synchronously moved (same speed) with the deforming circular region, a pixel of an image on the inside of the circular region was deformed at constant magnitude by the motion vector across frames. “Pixel warp” refers to a pixel warp method (Glasbey and Mardia, 1998). “Spatial Tukey window” refers to a two-dimensional spatial window to restrict the visibility of a stimulus image in a peripheral image area. The window consists of a combination of a rectangular window with a cosine window; the rectangular window was applied to keep the original image contrast on the inside of a central circular area with a diameter of 1.92 deg, while the cosine window gradually reduced the contrast in the peripheral area. **(D)** Some example images demonstrating how the appearance of the contour shape varies with the magnitude of contour deformation.

Stimuli contained a horizontally or vertically translating circular region wherein an image of a natural scene was non-rigidly deformed (Figures 1, 2, Supplementary Video 1). Ten natural images from the McGill Calibrated Color Image Database (Olmos and Kingdom, 2004) were used as the stimuli background (Figure 1). We first chose the translation direction of the circular region from four alternatives: upward, downward, leftward, and rightward. A water drop naturally slides on a window surface from top to bottom while a solid material can translate in various directions. Thus, it might be interesting to check whether the percepts of a material could be influenced by its translation direction. In particular, we were interested in whether the downward translation biased the percepts of a material to fluid more than the translations in other directions. The initial center positions were located at 1.8 deg (of visual angle) below and above the center of the stimulus image for the upward and downward conditions and to the right and left of it in the leftward and rightward conditions. The translation speed was 0.03 deg per frame (16.7 ms) and thus 1.8 deg/s. The temporal length of the stimuli was 2 s. We also controlled the magnitude of non-rigid deformation applied to the contour of the deforming region. Stimulus image generation had two stages: a contour deformation stage and an image deformation stage. (1) Contour deformation stage. We first created a sequence of translating binary circular region images (Figure 2A). The radius of the circular region was 1.92 deg. The binary image was deformed on the basis of horizontal and a vertical deformation maps that did not change within a sequence. The deformation map consisted of motion vector values by which the pixel values in the circular region image were shifted. The maps came from two-dimensional white noise images that were low-pass filtered with the cut-off frequency of 1.04 cpd, a value determined on the basis of the previous study (Kawabe et al., 2015a). We normalized the values of the deformation map so as to have absolute maximum values of 0, 0.18, 0.36, 0.54, 0.72, or 0.90 deg [see the resultant appearance of the deforming (and non-deforming) circular regions in Figure 2D]. A conventional inverse pixel warp method (Glasbey and Mardia, 1998) was used to deform the contour shape. (2) Image deformation stage. Within the (deforming or undeforming) circular region, the background image was deformed (Figures 2B,C). As we did for the circular region image, we created two sets of deformation maps for horizontal and vertical deformations. The image processing to create the deformation maps was identical to the one used in the contour deformation stage. The absolute maximum magnitude of image deformation was fixed at 0.36 deg. As shown in Figure 2B, the maps had effective regions that had shapes identical to the deformed circular regions on corresponding frames (please compare Figures 2A,B). The pattern on the inside of the deformed circular regions moved synchronously with the circular regions. That is, the spatial pattern of image deformation on the inside of the circular region was constant across frames. By using the deformation maps (Figure 2B), the background image was deformed by using the pixel warp method as was done in the contour deformation stage (Figure 2C). The spatial Tukey window (see the caption of Figure 2C) was applied to reduce the visibility of the peripheral image area to reduce artifacts due to deformation in the area.

#### Procedure

The experiments were conducted in a dimly lit room. Each observer was tested individually, and sat at 60 cm from the CRT display. On each trial, a neutral gray blank field was initially presented for 1 s, followed by the stimulus image sequence. The sequence, consisting of 120 images, lasted for 2 s. After the disappearance of the image sequence, a neutral gray blank field was again presented. The observers were asked to carefully view the image sequence, and after its disappearance, rate perceived material category on a 5-point scale: “1” for a solid material, “5” for a fluid material, and intermediate values for intermediate impressions, with an explicit instruction that “3” meant the material category was vague. Each session consisted of 48 trials consisting of 4 (translation directions) × 6 (levels of contour deformation magnitudes) × 2 (repetitions); in each trial the background image was randomly selected from among the 10 images previously chosen from the image database. Each observer experienced four sessions, and so it took ~40 min to complete all four. The order of trials was pseudo-randomized across the sessions within an observer, and across observers.

### Results and discussion

**Figure 3:**
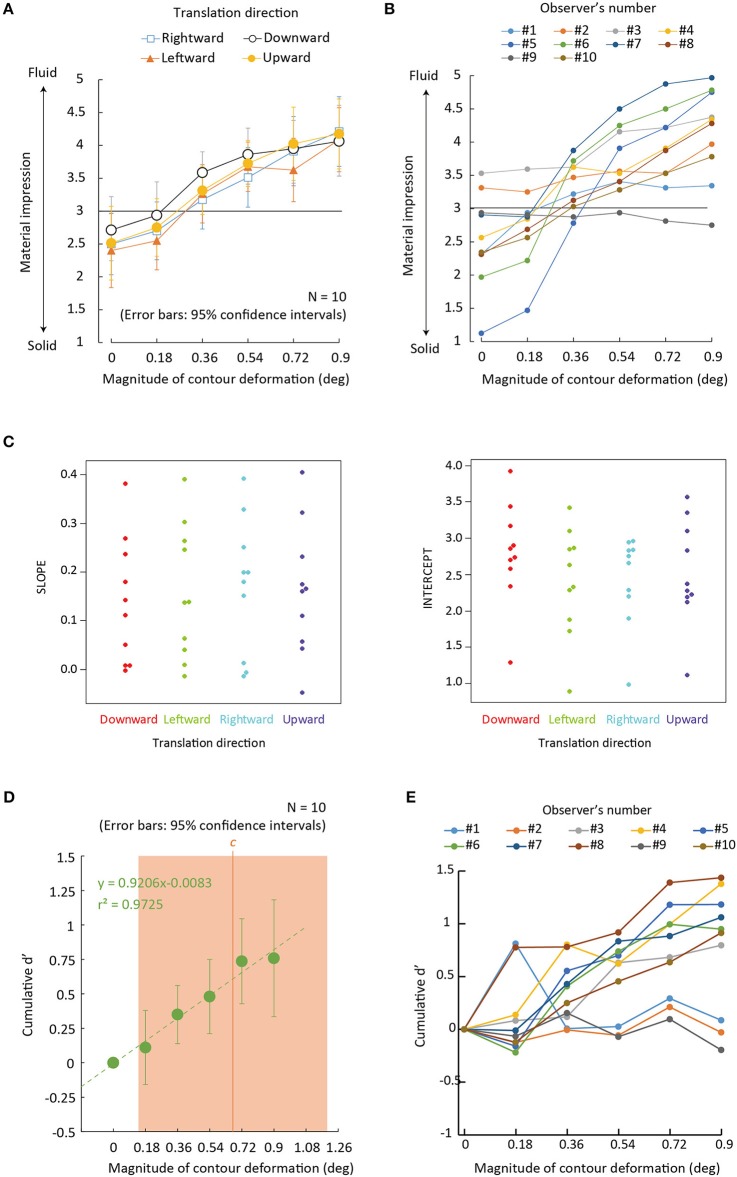
Experiment 1 results. **(A)** Average rating of material impressions between solid (1) and fluid (5) as a function of the magnitude of contour deformation for each translation direction. Error bars denote 95% confidence intervals (*N* = 10). **(B)** Individual data of **(A)** wherein the condition of translation direction is collapsed. **(C)** Beeswarm plots for the Individual slopes and intercepts of a linear function fitted to rating values as a function of the magnitude of contour deformations for each translation direction. **(D)** Green dots denote cumulative *d*′-value as a function of the magnitude of image deformation with 95 confidence intervals around the mean cumulative *d*′ for each level of contour deformation, collapsed across translation directions (*N* = 10). An orange vertical line denotes a decision criterion c between rating scores of “3” and “4.” An orange rectangular region denotes 95% confidence intervals around the mean c for each level of contour deformation, collapsed across translation directions (*N* = 10). **(E)** Individual cumulative *d*′ as a function of the magnitude of contour deformation.

For each observer, the rating scores for the material impressions were averaged across repetitions. Mean rating scores across the observers are plotted in Figure 3A for each translation direction as a function of the magnitude of contour deformation. Figure 3B also shows individual data wherein the data is collapsed across the four conditions of translation direction. To check whether the manipulation of the magnitude of contour deformation biased the percept of a material, we calculated 95% confidence intervals around the mean rating score for each level of contour deformation. By using the Holm–Bonferroni test (*p* < 0.05), we assessed whether the mean rating score for each level of contour deformation was statistically deviated from the value of “3” (the vague impression). The results showed that in all translation direction conditions, the rating scores were significantly greater than “3” when the magnitude of contour deformation was 0.90 deg (see Table 1 for exact *p*-values). In all translation direction conditions except the leftward condition, the rating scores were significantly greater than “3” when the magnitude of contour deformation was 0.72 deg. In the downward condition, the rating scores were significantly greater than “3” when the magnitude of contour deformation was 0.54 deg. On the other hand, the rating scores were not significantly lower than “3” in all of the translation direction conditions.

**Table 1:** The results of multiple comparison tests with the Holm–Bonferroni test between the mean rating score for each level of contour deformation and the value of “3” (the vague impression) in Experiment 1.

**The magnitude of contour deformation**	**Translation direction**	***t***	**Adjusted *p***
0	Rightward	2.42	1.000
	Leftward	2.41	0.277
	Downward	1.28	1.000
	Upward	1.97	0.648
0.18	Rightward	1.54	1.000
	Leftward	2.29	0.748
	Downward	0.28	1.000
	Upward	1.29	1.000
0.36	Rightward	0.89	1.000
	Leftward	1.34	1.000
	Downward	4.21	0.306
	Upward	1.95	1.000
0.54	Rightward	2.56	0.621
	Leftward	4.07	0.172
	Downward	4.87	0.022
	Upward	4.69	0.115
0.72	Rightward	3.92	0.022
	Leftward	2.93	0.247
	Downward	4.99	0.010
	Upward	4.16	0.006
0.9	Rightward	5.18	<0.001
	Leftward	5.06	0.003
	Downward	4.38	0.003
	Upward	5.00	0.001

*Adjusted p-values are reported*.

To check the statistical significance of the effect of translation direction on the rating scores, we assessed the slope and intercept of a linear function fitted to rating scores as a function of the magnitude of contour deformation (Figure 3C). The slope was tested in order to examine whether the sensitivity of the rating to the magnitude of contour deformation was dependent on the translation direction. The intercept was assessed in order to examine whether there was any bias of the rating that depended on the translation directions. For the two indices, we separately conducted a one-way repeated measures ANOVA with translation direction as a within-subject factor. The results showed no significant main effect was obtained for the slope [*F*_(3, 27)_ = 2.286, *p* = 0.104, partial η^2^ = 0.2]. The main effect was significant for the intercept [*F*_(3, 27)_ = 5.709, *p* = 0.0037, partial η^2^ = 0.38]. Multiple comparison tests (Ryan, 1959) showed that the rating scores in the downward condition had the higher intercept than the other three conditions (*p* < 0.03). The results indicate that the downward translation biases the percept of deforming regions toward liquid.

To check to what extent the perceptual dimension of “liquidness” was affected by contour deformation, on the basis of signal detection theory (Green and Swets, 1966), we calculated a cumulative *d*′-value as a function of the magnitude of contour deformation by means of the procedure as described in the previous study (MacMillan and Creelman, 2005, Chapters 3, 5). Specifically, we tabulated the frequency of reporting each rating score for each magnitude of contour deformation. Here we collapsed the data across translation directions. For each adjacent pair of conditions of the magnitude of contour deformation, we calculated *d*′ that was the measurement for the sensitivity to a signal among a noise. Because four estimates (between ratings 1 and 2, 2 and 3, 3 and 4, and 4 and 5) were available for each magnitude of contour deformation, we averaged them. Figure 3D shows the cumulative *d*′-values with 95% confidence intervals around the mean cumulative *d*′ for each level of contour deformation, collapsed across translation directions. We conducted a two-tailed one-sample *t*-test to check whether the cumulative *d*′ for each level of contour deformation was statistically different from zero. The cumulative *d*′ was not significantly deviated from 0 when the magnitude of contour deformation was 0.18 deg [*t*_(9)_ = 0.931, *p* = 0.3762] while it was significantly higher than 0 when the magnitudes of contour deformation were 0.36 deg [*t*_(9)_ = 3.740, *p* = 0.0046], 0.54 deg [*t*_(9)_ = 4.034, *p* = 0.003], 0.72 deg [*t*_(9)_ = 5.396, *p* < 0.001], and 0.90 deg [*t*_(9)_ = 4.047, *p* = 0.0029]. We also checked decision criteria *c*. Values above and below *c* lead to the response “signal is present” and “signal is absent,” respectively. We found that *c* (0.66 ± 0.52 for mean *c value* ±95% confidence interval) for yielding rating scores between 3 and 4 was included in the range of our stimulus manipulation, but not for *c*s between 1 and 2 (−0.93 ± 0.24), between 2 and 3 (−0.3 ± 0.29), and between 4 and 5 (1.233 ± 0.46). Figure 3E shows the individual data of the cumulative *d*′-values. Clearly, there were a couple of participants who showed no effect of contour deformation. The individual difference may lead to the large confidence intervals for *d*′ and *c*, and may make the statistical analyses sensitive to outliers.

To sum, the results showed that material categorization was biased toward liquid when non-rigid deformation greater than at least 0.54 deg was given to the contour of a deforming region. This result indicates that the non-rigid deformation of the contour of a deforming region serves as a visual clue that biases the percept of a material toward liquid.

As shown in Figure 3B, some observers did not respond to the magnitude of contour deformation. There are several possible reasons for this. One possibility is that these observers did not notice the variation of the contour shape, and made their assessments solely on the pattern of image deformation on the inside of the circular region. The other possibility is that there is an inherent individual difference in using visual information when judging material impression. Specifically, those who did not respond to the magnitude of contour deformation might not use contour shape as useful information in determining the material impression of a deforming area. Future studies need to address this issue, for example, by manipulating the state of an observer's attention so as to focus on the dynamic change in contour shape.

## Experiment 2

### Purpose

The purpose of this experiment was to explore whether spatially blurring the contour of a deforming region biased the percept of the region toward liquid. In Experiment 1, we found that a liquid impression was invoked when the contour of a deforming region was non-rigidly deformed to a significant extent. The results motivated us to investigate whether the lack of the rigid contour structure biased the percept of a deforming region toward liquid even when non-rigid deformation was not explicitly given to a contour. As described earlier, spatially blurring the contour of a gray patch against a textured background produces the percept of fog or smoke, which are non-rigid materials (Anderson et al., 2006). We explored whether spatially blurring the contour of a deforming region biased the percept of a deforming region toward liquid; this was achieved by manipulating the standard deviation of the Gaussian filter applied to the contour of the region.

### Method

#### Observers

Ten observers, who had participated in Experiment 1, participated in this experiment. They were still unaware of the purpose of the experiment.

#### Stimuli

Stimuli were identical to those as used in Experiment 1 except for the following. Instead of the magnitude of contour deformation, we manipulated the standard deviation (*SD*) of the Gaussian blur filter that was applied to a circular region at six levels: 0, 0.06, 0.12, 0.18, 0.24, and 0.3 deg (Supplementary Video 2). Here, the 0 deg *SD* condition was just identical to the 0 deg condition in Experiment 1.

#### Procedure

The procedure was also identical to that as used in Experiment 1 except for the following. Each session consisted of 48 trials consisting of 4 (translation directions) × 6 (levels of the *SD* of the Gaussian filter) × 2 (repetitions). Each observer received four sessions, and so it took ~40 min to complete all four.

### Results and discussion

**Figure 4:**
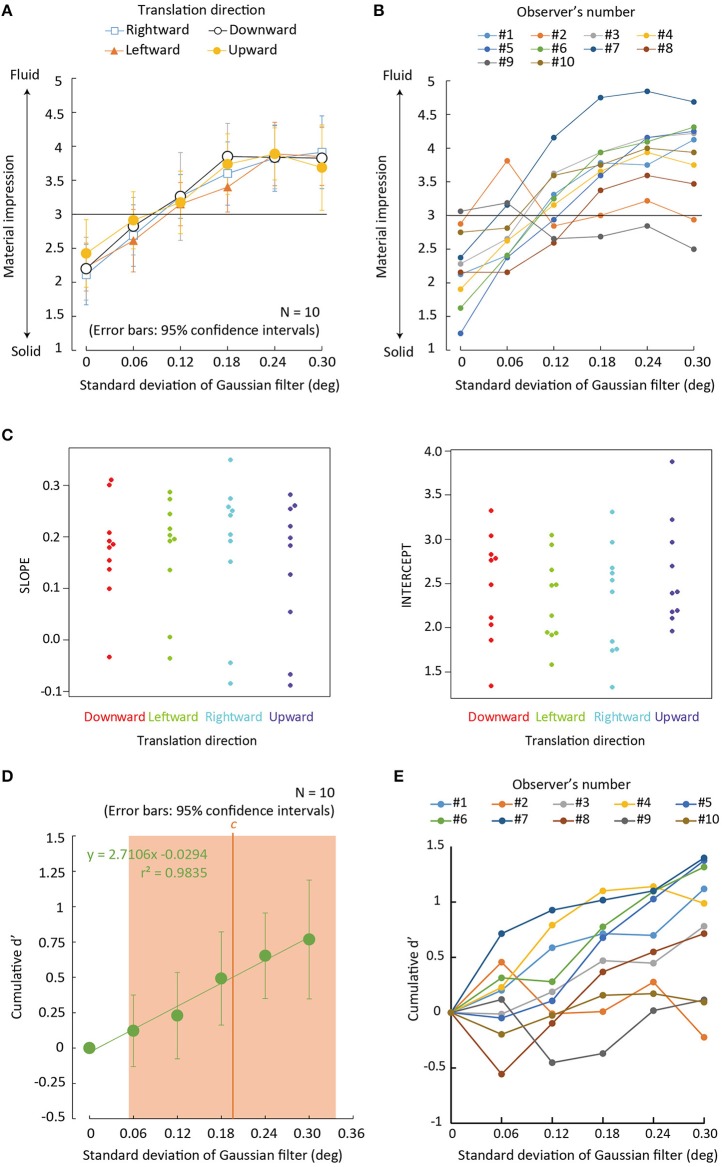
Experiment 2 results. **(A)** Average rating of material impressions between solid (1) and fluid (5) as a function of the *SD* of the Gaussian blur filter that was applied to the contour of a deforming region. Error bars denote 95% confidence intervals (*N* = 10). **(B)** Individual data of **(A)** wherein the condition of a translation direction is collapsed. **(C)** Beeswarm plots for the Individual slopes and intercepts of a linear function fitted to rating values as a function of the magnitude of the *SD* of Gaussian filter for each translation direction. **(D)** Green dots denote cumulative *d*′-value as a function of the magnitude of image deformation with 95 confidence intervals around the mean cumulative *d*′ for each level of contour deformation, collapsed across translation directions (*N* = 10). An orange vertical line denotes a decision criterion c between rating scores of “3” and “4.” An orange rectangular region denotes 95% confidence intervals of around the mean c for each level of the *SD*, collapsed across translation directions (*N* = 10). **(E)** Individual cumulative *d*′ as a function of the *SD* of the Gaussian blur filter.

For each observer, the rating scores for the material impressions were averaged across repetitions. Mean rating scores across the observers are plotted in Figure 4A for each translation direction as a function of the *SD* of the Gaussian filter. Figure 4B also shows individual data wherein the data is collapsed across the four conditions of translation direction. To check whether the manipulation of the *SD* biased the percepts of a material, we calculated 95% confidence intervals around the mean rating score for each level of the *SD* of the Gaussian filter. By using the Holm–Bonferroni test (*p* < 0.05), we assessed whether the mean rating score for each level of contour deformation was statistically deviated from the value of “3” (the vague impression). The results showed that the rating scores were significantly larger than “3” when the *SD* was 0.24 deg in all translation direction conditions except the downward condition, and when the *SD* was 0.3 deg in the rightward and leftward conditions (see Table 2 for exact *p*-values). Moreover, the rating scores was significantly lower than “3” when the *SD* was zero in all translation direction conditions except the downward condition.

**Table 2:** The results of multiple comparison tests with the Holm–Bonferroni test between the mean rating score for each level of contour deformation and the value of “3” (the vague impression) in Experiment 2.

**The magnitude of contour deformation**	**Translation direction**	***t***	***p***
0	Rightward	4.53	0.017
	Leftward	4.98	0.017
	Downward	3.92	0.076
	Upward	2.62	0.420
0.06	Rightward	1.56	1.000
	Leftward	1.91	0.542
	Downward	0.94	1.000
	Upward	0.47	1.000
0.12	Rightward	1.84	1.000
	Leftward	1.07	1.000
	Downward	0.92	1.000
	Upward	0.86	1.000
0.18	Rightward	2.92	0.262
	Leftward	2.47	0.542
	Downward	3.96	0.062
	Upward	3.75	0.128
0.24	Rightward	3.84	0.032
	Leftward	4.30	0.005
	Downward	4.10	0.065
	Upward	5.25	0.036
0.3	Rightward	3.86	0.015
	Leftward	4.52	0.007
	Downward	3.96	0.067
	Upward	2.47	0.173

*Adjusted p-values are reported*.

To check the statistical significance of the effect of translation direction on the rating scores, we assessed the slope and intercept of a linear function fitted to rating scores as a function of the magnitude of contour deformation (Figure 4C). For the two indices, we separately conducted a one-way repeated measures ANOVA with translation direction as a within-subject factor. The results showed no significant main effect was obtained for the slope [*F*_(3, 27)_ = 1.478, *p* = 0.2428, partial η^2^ = 0.14]. The main effect was not also significant for the intercept [*F*_(3, 27)_ = 2.313, *p* = 0.0985, partial η^2^ = 0.20]. Inconsistent with the results of Experiment 1, the results of this experiment showed no higher bias in the downward than other conditions for perceiving deforming regions toward liquid. At this stage it is unclear why there was the difference in the bias of the downward condition between the experiments. There are several possible explanations for the difference. First, the effect of translation directions on the bias toward a liquid impression may appear only when the contour of deforming regions is deformed. Second, because the observers had already participated in Experiment 1 and learned the statistics of the stimuli in the experiment, the natural bias for the downward translation may have been reduced in this experiment. Finally, the bias is possibly small and given that the number of repetitions at each blur was very low, the noise in the data might drown out the effect. It is necessary for future studies to closely address these possibilities.

In addition, to check to what extent the perceptual dimension of “liquidness” was affected by a contour deformation, we calculated a cumulative *d*′-value as a function of the *SD*. Figure 4D shows the cumulative *d*′-value with 95% confidence intervals around the mean cumulative *d*′ for each level of contour deformation, collapsed across translation directions. We conducted a two-tailed one-sample *t*-test to check whether the cumulative *d*′ for each level of the *SD* of the Gaussian blur filter was statistically different from zero. The cumulative *d*′ was not significantly deviated from zero when the *SD* was 0.06 deg [*t*_(9)_ = 1.091, *p* = 0.304] and 0.12 deg [*t*_(9)_ = 1.699, *p* = 0.124]. Meanwhile, the cumulative *d*′ was significantly higher than zero when the *SD* was 0.18 deg [*t*_(9)_ = 3.372, *p* = 0.008], 0.24 deg [*t*_(9)_ = 4.886, *p* < 0.001], and 0.30 deg [*t*_(9)_ = 4.135, *p* = 0.003]. We also found that *c* (0.195 ± 0.141 for mean *c* ± 95% confidence interval) for determining the rating scores between 3 and 4 was included in the range of our stimulus manipulation, but not for *c*s between 1 and 2 (−0.34 ± 0.037), between 2 and 3 (−0.09 ± 0.093), and between 4 and 5 (0.537 ± 0.137). Figure 4E shows the individual data of the cumulative *d*′-values. Similarly to Experiment 1 (Figure 3E), there were a couple of participants who showed no effect of the Gaussian blur filter applied to the contour of deforming regions. The individual difference may again lead to the large confidence intervals for *d*′ and *c*, and may make the statistical analyses sensitive to outliers.

The results indicate that spatially blurring of the contour of a deforming region biases the percept of the region toward liquid in a moderate manner. In accordance with the effect of image blurring in a luminance contrast dimension (Anderson et al., 2006), spatially blurring the contour of a deforming region also possibly biases the percept of the region toward a non-rigid material, in this case, liquid.

In this experiment, the rating scores in the 0 deg condition were significantly deviated from “3” while those in the 0 deg condition were not in Experiment 1, though both conditions involved identical stimulus. By using a two-tailed *t*-test, we compared the rating scores collapsed across translation directions between the 0 deg conditions of the two experiments, but did not acknowledge a significant difference [*t*_(9)_ = 1.951, *p* > 0.8]. Hence, we would like to conclude that there was no essential difference in the rating values of the 0 deg conditions between the experiments. As shown by the analysis of *c*, no decision boundary for a solid material impression existed in the range of our stimulus manipulations. Thus, we suggest it is safe to conclude that our stimulus manipulation did not strongly contribute to the formation of a solid material impression.

## General discussion

We examined how the non-rigidity of the contour of a deforming region biased the percept of the region toward liquid. In Experiment 1, we observed that the large contour deformation reliably biased the percept toward liquid. In Experiment 2, we showed that spatially blurring the contour of a deforming region biased the percept toward liquid.

Our study successfully showed that the non-rigid deformation of the contour of a deforming region biased the percept toward liquid. In our stimuli, the pattern of image deformation within a deforming region rigidly moved along its trajectory in all conditions. Thus, the visual system possibly used the non-rigid deformation and/or the spatial blur of the contour of a deforming region to interpret the rigidly moving image deformation. The results are consistent with previous studies proposing that the information from the contour is used to perceptually determine the shape and/or material properties of the surface (Knill, 1992; Nefs, 2008; Todorović, 2014; Marlow and Anderson, 2015; Marlow et al., 2015).

A link between the perception of non-rigid contours and the perception of liquid was shown in the present study. Previous studies have reported several image features such as shape (Paulun et al., 2016; van Assen and Fleming, 2016), motion (Kawabe et al., 2015b), and image deformations at surfaces (Kawabe et al., 2015a; Kawabe and Kogovšek, 2017) caused the perception of a liquid material. In addition to these image features, the present study indicates that contour non-rigidity is perceptually linked to a liquid material. The perception of contour non-rigidity is affected by shape properties such as symmetry (Cohen et al., 2010). It would be intriguing to examine how shape properties affect the perception of liquid. Moreover, because our manipulation of contour deformation was conducted with a limited range of spatiotemporal frequency, it is necessary to check what sort of material impression is reported when the contour is deformed at the different ranges of spatiotemporal frequency. For example, an elastic material, which also likely causes non-rigid contour deformation, produces oscillative image deformation patterns (Masuda et al., 2013; Kawabe and Nishida, 2016). It is necessary to check how the percept is biased when oscillative deformation is given to the contour of a deforming region.

As a precursor, we tested how the shape complexity of the contour of a deforming region influenced the impression of solid and liquid materials. Supplementary Video 3 shows a clip wherein a deforming and/or intact cross or star is spatially translated. With the video clip, we observed that the intact cross and star produced a highly rigid and solid-like material impression while the deforming cross and star produced a non-rigid and liquid-like material impression. It was possible that the shape complexity created by the non-rigid image deformation could have been the source of liquid impression in Experiment 1. However, Supplementary Video 3 showed that the cross and star, which were more complex than the circle used in Experiment 1, did not produce liquid impression unless a deformation was added to the contour of a deforming region. Although, formal examinations are necessary, we suggest that shape complexity itself may not contribute to liquid impressions. Systematic manipulations of shape complexity and contour deformation magnitudes will be required to check how those two factors intact with each other.

We additionally addressed how the occlusion of the contour of a deforming region had an effect of material appearance (Supplementary Video 4). We conducted a preliminary observation with two naive observers. They reported that the deforming region with an occluder was perceived to be more rigid than the deforming region without the occluder. However, an additional observer who watched the video clip reported that the deforming region with the occluder seemed more liquid that the one with the 0 deg contour deformation. We suggest that the discrepancy of appearance among the observers may stem from the observers' “set” that possibly determines the part on which the observers focus in the clip. For the observers focusing on the occluder's motion, the rigid motion signal of the translating occluder helped the observers to perceive the rigid component in the translation of deforming regions, and percepts in the observers would be biased toward solid. On the other hand, for the observers focusing on the interior information, the occluder serves to hide information of the surface contour, and percepts would be biased toward liquid. Thus, for clarifying the set by observers, it is interesting to manipulate the presence/absence of occluders, and test what the rating would be.

Another interpretation of the results in Experiment 2 is that spatially blurring the contour of a deforming region caused the spatial gradient in the magnitude of image deformation across space, and the spatial gradient in the magnitude of image deformation caused the spatial gradient of a perceived material's thickness across space. The magnitude of image deformation influences the perceived thickness of transparent materials (Fleming et al., 2011). Hence, the thickness gradient caused by spatial blur might be perceptually linked to the curved surface of a liquid material. When the spatial blur is not applied to a deforming region, perceptual thickness does not change across space and time. The constant thickness perception might not bias the percept into liquid.

In both experiments, the observers rarely reported a solid material impression though in the 0 deg condition of Experiment 2 the rating scores were significantly deviated from 3. As a result of an analysis based on signal detection theory, we found that only the decision boundary between ratings 3 and 4 existed on the inside of the range of our stimulus manipulation. The results indicate that our stimulus manipulation contributed to biasing the percept toward liquid, but did not contribute to biasing the percept toward solid. There are several possible reasons why our stimuli did not invoke the impression of a solid material. First, because the spatial deformation patterns in a deforming region were complex, the observer was reluctant to give a rating value favoring the impression of a solid material even when the contour of the region was not deformed. Usually, the surface of a solid material is smooth, and rarely ripples unlike the surfaces of liquids. Because the spatial frequency of our image deformation was determined so that it was optimal for transparent liquid perception (Kawabe et al., 2015a), the interpretation on the basis of a contour rigidity might contradict the interpretation made on the basis of spatial frequency of image deformation, and consequently the impression might be ambiguous. There was also a possibility that the observers actually felt the variation of liquid “viscosity,” instead of the variation of material categories, as a function of our stimulus manipulation though we asked them to report the impression of solid and liquid materials. Due to this, the observer might interpret stimuli with no deforming contour as a material having a high viscosity, and occasionally report rating values around 3 because the observers were unsure of whether such high viscosity materials should be reported as liquid or solid. Future studies need to carefully check both the role of deformation spatial frequency and the role of rating criteria in the determination of material perception on the basis of image deformation.

## Author contributions

TK designed and conducted the experiments, analyzed the data, and wrote the manuscript.

### Conflict of interest statement

TK is an employee of NTT Communication Science Laboratories, which is a basic-science research section of Nippon Telegraph and Telecommunication corporation (NTT). There is a pending patent involving the reported research. There are no products in development or marketed products to declare. The pending patent does not alter the author's adherence to policies of Frontiers.
